# Feasibility of e-commerce pharmacy provision and acceptability of levonorgestrel 1.5 mg for pericoital use in urban and peri-urban settings in Kenya: a prospective cohort study

**DOI:** 10.1136/bmjsrh-2022-201653

**Published:** 2022-11-02

**Authors:** George Odwe, Brent McCann, Wilson Liambila, Jessica Vandermark, Daniel Mwanga, Edna Anab, Moses Wanami, Saumya Ramarao

**Affiliations:** 1 Reproductive Health, Population Council - Kenya, Nairobi, Kenya; 2 Camber Collective, Seattle, Washington, USA; 3 Data Science and Evaluation, African Population and Health Research Center, Nairobi, Kenya; 4 Kasha, Nairobi, Kenya; 5 Independent Consultant, Nairobi, Kenya; 6 Reproductive Health, Population Council Inc, New York City, New York, USA

**Keywords:** contraception behavior, contraceptives, oral, contraceptives, postcoital, Contraceptive Effectiveness

## Abstract

**Introduction:**

An ‘on-demand’ contraceptive pill may suit women having infrequent sex. We assessed the feasibility of e-commerce pharmacy provision and acceptability of levonorgestrel (LNG) 1.5 mg for pericoital use in Kenya.

**Methods:**

A 12-month prospective, single-arm, open-label, interventional study conducted from August 2020 to August 2021. A total of 897 women aged 18–49 years at risk of pregnancy and having infrequent sex (ie, six times or fewer coital frequency/month) were screened and enrolled. We undertook univariate and bivariate analysis on multiple data points: enrolment dataset, bimonthly surveys, extended survey around 6-month follow-up, and e-commerce pharmacy sales log.

**Results:**

A total of 2291 pericoital LNG 1.5 mg pill doses were dispensed to 300 women over a 12-month period mainly via the USSD (Unstructured Supplementary Service Data) platform. Of the 266 women who obtained the pill and completed the survey, most (83%) were satisfied/very satisfied with e-commerce platform services, citing convenience and ease of use. The pill was also acceptable. Of the 266 women who used it at least once, 94% were satisfied/very satisfied, likely to continue using and would recommend it to others; 86% used it within 24 hours before/after sex as recommended and most of the time they had sex; and 147 (55%) experienced side effects, mainly nausea, headache, changes in menstrual pattern, cramps and dizziness that were mild and tolerable.

**Conclusions:**

E-commerce pharmacy provision of LNG 1.5 mg for pericoital use is feasible. In addition, a pericoital LNG 1.5 mg pill is acceptable among women having infrequent sex and could potentially address their unmet family planning needs.

**What is already known on this topic**

- Levonorgestrel (LNG) 1.5 mg, a progestogen-only emergency contraceptive pill, is a potential method women can use on-demand or pericoitally (ie, within 24 hours before or after sex).
- Evidence on feasibility of e-commerce pharmacy provision and acceptability of LNG 1.5 mg for pericoital use in low- and middle-income counties is scarce.

**What this study adds**

- E-commerce pharmacy provision of LNG 1.5 mg for pericoital use is feasible.
- LNG 1.5 mg for pericoital use is acceptable as a primary contraceptive method among women having infrequent sex.

**How this study might affect research, practice or policy**

- Adding the pericoital LNG 1.5 mg pill to the family planning method mix could expand method choice and address unmet need for family planning among women having infrequent sex including those who have never used, are dissatisfied or discontinued other modern methods.

## Introduction

In 2019, an estimated 218 million women of reproductive age in low- and middle-income countries (LMICs) had an unmet need for family planning (FP) (ie, sexually active and wanted to avoid becoming pregnant but were not using contraception).^1^ Many of these women cite concerns about health, side effects, and infrequent sexual activity as the main reasons for not using contraception.^2^ While Kenya has made remarkable progress in modern contraceptive prevalence rate (CPR) from 39.4% in 2008/09 to 53.2% in 2014, contraceptive discontinuation is common due to dissatisfaction with current methods.^3^ Furthermore, about one in five (18%) of currently married women in Kenya have an unmet need for FP, mainly due to health concerns, fear of side effects, and infrequent sex/no sex.^4^

Women having infrequent sex may be reluctant to take daily pills or use long-term methods due to intermittent risk of pregnancy or concerns about health risks such as interference with menstrual bleeding and infertility.^5^ An on-demand contraceptive pill may assuage these reasons due to the possibility of high compliance/adherence and covert use before/after sex without the cooperation or the knowledge of a partner.^5 6^ Furthermore, an on-demand contraceptive pill may be acceptable among young and unmarried women who may have diminished decision-making power or face cost or social barriers to accessing modern contraceptives.^2 7^

Levonorgestrel (LNG), a progestogen-only emergency contraceptive pill (ECP), is a potential method women can use on-demand or pericoitally (ie, within 24 hours before or after coitus).^8–11^ LNG is currently distributed globally in a single dose of 1.5 mg or a double dose of 0.75 mg pill, which is recommended to be taken for emergency use only, within 5 days after unprotected sexual intercourse.^12^ Extensive research on LNG, including repeat use, dates to the beginning of the 1970s. LNG pills were originally labelled in Eastern Europe and Asia in the 1980s and 1990s as a contraceptive to be taken before or after sex, as needed.^9^ Recent studies have assessed pericoital use of LNG 1.5 mg pill as a primary means of contraception.^10 13^ Single or repeated use of LNG by women and adolescents have been shown to be safe, efficacious and effective.^14–17^ Evidence also indicates that repeated pericoital use of LNG 1.5 mg is feasible and acceptable.^9 10 14^ However, there is scarce evidence on the acceptability and safety of LNG 1.5 mg for pericoital use in LMICs.

This study assessed the feasibility of e-commerce provision and acceptability of LNG 1.5 mg for pericoital use as a primary contraceptive method based on an intervention study in two urban and peri-urban settings—Nairobi and Kiambu counties in Kenya. The findings can inform policy and programme discussions around the potential of LNG 1.5 mg for pericoital use as an additional contraceptive choice.

## Methods

### Study design and procedures

The study was a 12-month prospective, single-arm, open-label, interventional study conducted in urban and peri-urban areas of Nairobi and Kiambu counties from August 2020 to August 2021. The intervention consisted of screening and recruitment of eligible women of reproductive age and distribution of pericoital LNG 1.5 mg contraceptive pill (study pill) through Kasha—an e-commerce pharmacy platform distributing health, hygiene and self-care products in Kenya. Nairobi and Kiambu counties were selected due to relatively high modern CPR at 58% and 68%, respectively, and are currently served by Kasha distribution network that allows same day or next day delivery. Women were recruited from (a) 20 health facilities (12 in Nairobi County and 8 in Kiambu County including public dispensaries/health centres and privately run clinics) by health providers working at FP service unit (ie, nurses, clinical officers) and (b) in the community surrounding these facilities by trained community health volunteers (CHVs). CHVs are community members selected and trained to provide basic health information and services, including FP information, in the areas they live.^18^ Women seeking FP services were counselled on full contraceptive choice, including LNG 1.5 mg for pericoital use (the study pill) by health providers and CHVs during routine household visits. If interested, women called a toll-free number to speak to a trained female research assistant who provided further details about the study. Women were screened for eligibility via telephone and enrolled if eligible. An automatic SMS (short message service) with a unique and anonymous ID number was generated and sent to the participant’s mobile number to confirm enrolment and provide instructions on obtaining the study pill from the e-commerce pharmacy platform should she need it.

Women were eligible if they were aged 18–49 years, had infrequent sex (ie, said that they had sex six times or fewer in a month), were not currently pregnant or using a modern method (except condoms due to their protection against sexually transmitted infections (STIs)/HIV), and did not wish to get pregnant during the study period but would not consider it a serious issue. We used the World Health Organization (WHO)-endorsed pregnancy checklist to assess pregnancy at enrolment. In addition, women who reported having pre-existing conditions (ie, HIV/AIDS, hypertension, diabetes) were excluded.

The study pill was only available via Kasha e-pharmacy. Women could order the study pill directly online through Kasha’s website, USSD (Unstructured Supplementary Service Data) or a call centre, using a unique enrolment ID obtained at enrolment. On ordering, women were counselled via telephone by trained Kasha pharmacists on how to use the LNG 1.5 mg pill, namely to take only one pill within 24 hours before or after sexual intercourse, and no more than six times per month, and use LNG 1.5 mg for pericoital use as their exclusive method. The study pill was delivered to the study participant at her choice of location, typically her home, for free using Kasha’s direct-to-consumer (D2C) distribution system.

Enrolled participants were followed up every 2 weeks by telephone (bimonthly survey) until the end of the study (August 2021). Women were unenrolled from the study if they had become or wished to get pregnant, were no longer interested in participating, moved out of the study area permanently, or had switched/adopted a modern method (except condoms) during the study period.

The study received ethical approval from the Population Council IRB (Protocol 890), the African Medical and Research Foundation Ethics and Scientific Review Committee (AMREF-ESRC P619/2019) and regulatory approval from the Pharmacy and Poisons Board (PPB//ECCT/20/02/03/2021). In addition, all study participants provided verbal informed consent at recruitment and written confirmation at delivery of the first order of the study pill.

### Data collection and analysis

Women were interviewed via telephone at baseline (ie, screening data such as age, average sex per month and prior use of contraceptives) and twice a month (ie, bimonthly surveys) and once at the 6-month point (extended survey). The bimonthly surveys gathered information on the study pill use, satisfaction, and experience of side effects. The extended survey collected information on demographic background, contraceptives use, satisfaction with the study pill and services, and future contraceptive intentions. The e-commerce pharmacy sales registry dataset provided information on the quantity and doses dispensed.

We calculated a sample size of 768 (384/site) women with unmet need for contraceptives and having infrequent sex based on prevalence formulae with 95% confidence level and 5% margin of error.^19^ The sample size was increased to 900 assuming 15%–20% lost to follow-up.

*Feasibility* of e-commerce provision of LNG 1.5 mg for pericoital use was evaluated in terms of women’s ability to obtain the study pill from Kasha, and satisfaction with e-commerce pharmacy services. *Acceptability* of the pericoital LNG 1.5 mg pill was evaluated based on use of the study pill, self-reported adherence or compliance, satisfaction, the likelihood of future use and safety (self-reported side effects). We used descriptive and bivariate statistics with chi-square to test statistical significance differences between groups at p<0.05 and 95% confidence intervals. Data were analysed using Stata version 15.1.

## Results

### Background characteristics

**Figure 1:**
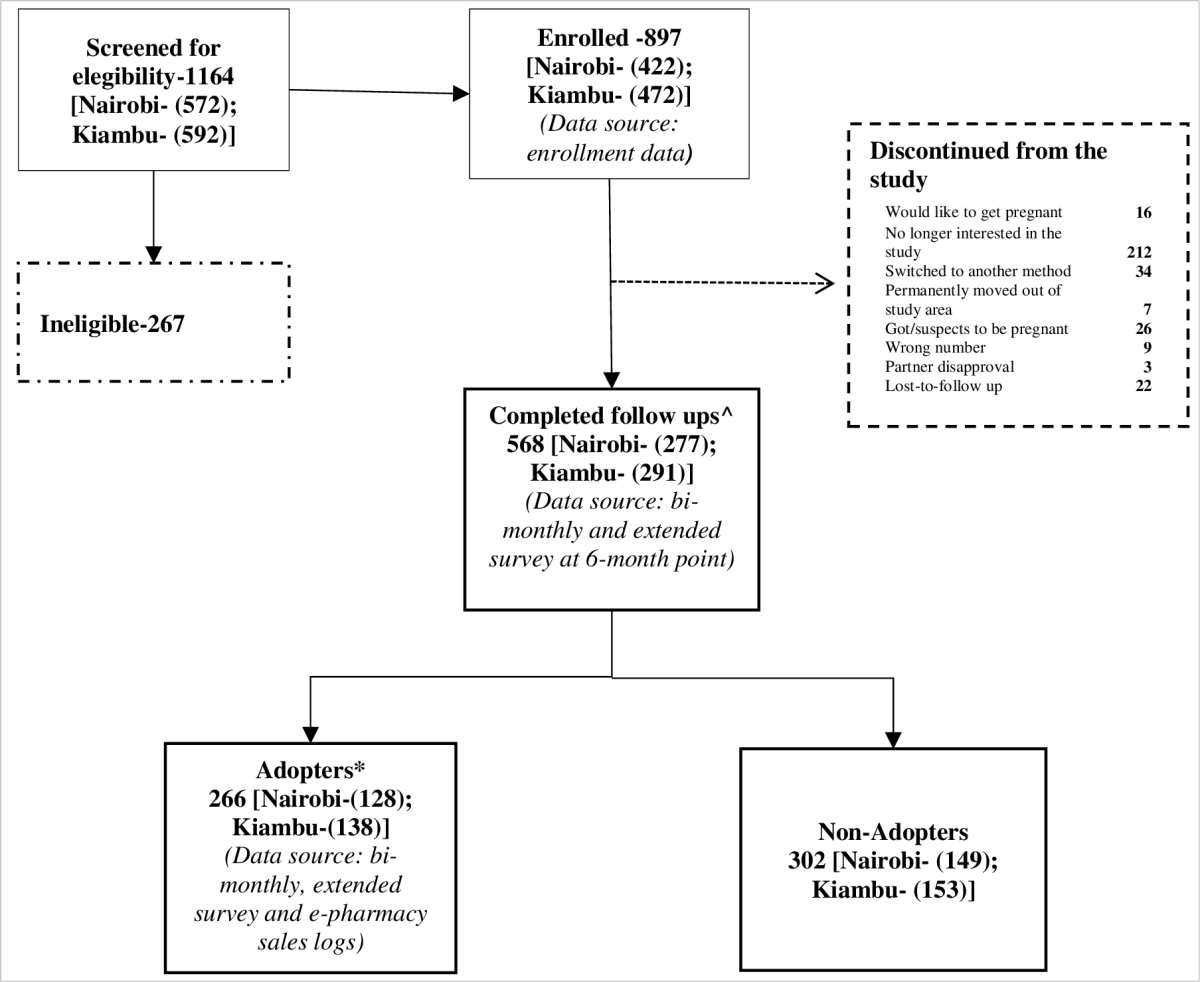
Participant flowchart for the study. ^∧^Completed both bimonthly survey and extended survey. *Defined as women who (1) obtained the study pill from Kasha’s e-commerce platform; (2) reported using the study pill at least once and (3) provided their demographic data via the extended surveys.

Of the 1164 women screened, 897 were eligible and enrolled in the study on consenting (figure 1). Of these, 568 (63%) women completed bimonthly and extended surveys. Among women who completed bimonthly and extended surveys, 266 (47%) reported obtaining and using LNG 1.5 mg for pericoital use at least once (adopters), while 302 (53%) did not obtain or report using the pill (non-adopters).

Most of the participants wanted a child or another child, had ever used a modern method or did not perceive it as a problem if they became pregnant. Adopters were more likely than non-adopters to have more children and used a modern method (ie, intrauterine device (IUD), injectable, implant, daily pill). However, there were no significant variations between adopters and non-adopters by sociodemographic characteristics (age, education and marital status) (table 1).

**Table 1:** Background characteristics of levonorgestrel 1.5 mg pericoital pill adopters and non-adopters

Characteristic	Adopters (n=266) (n (%))	Non-adopters (n=302) (n (%))	All (n=568) (n (%))	P value
Mean age (years) (SD)	26.4 (5)	25.6 (5)	25.9 (5)	0.098
Marital satus				
Single, never married	170 (63.9)	208 (68.9)	378 (66.5)	0.447
Married	87 (32.7)	86 (28.5)	173 (30.5)	
Divorced, widowed, separated	9 (3.4)	8 (2.7)	17 (3.0)	
Education level				
Primary/secondary incomplete	14 (5.3)	18 (6.0)	32 (5.6)	0.932
Secondary complete	116 (43.6)	132 (43.7)	248 (43.7)	
Higher	136 (51.1)	152 (50.3)	288 (50.7)	
Has a child/children (% yes)	147 (55.3)	137 (45.4)	284 (50.0)	0.019
Want a/another child (% yes)	220 (82.7)	249 (82.6)	469 (82.6)	0.975
If you were to find out that you were pregnant today, would this be a problem?				
Yes, a big problem	62 (23.3)	63 (20.9)	125 (22.0)	0.744
Yes, a small problem	53 (19.9)	59 (19.5)	112 (19.7)	
No, it would not be a problem	151 (56.7)	180 (59.6)	331 (58.3)	
Ever used a modern method	237 (89.1)	254 (84.1)	491 (86.4)	0.299
Type of method previously used*				
Emergency contraception	126 (47.4)	132 (43.7)	258 (45.4)	0.715
Male condom	117 (44.0)	137 (45.4)	254 (44.7)	0.359
Female condom	4 (1.5)	2 (0.7)	6 (1.1)	0.359
Pill	66 (24.8)	58 (19.2)	124 (21.8)	0.219
IUD	13 (4.9)	12 (4.0)	25 (4.4)	0.685
Injectable	54 (20.3)	39 (12.9)	93 (16.4)	0.042
Implant	45 (16.9)	35 (11.6)	80 (14.1)	0.109
Other	6 (2.3)	0 (0.0)	6 (1.1)	–
Proportion of women who ever used pills, injectables, implants or IUD	129 (48.5)	111 (36.9)	240 (42.3)	0.035
Place of residence				
Nairobi	128 (48.5)	149 (49.3)	277 (48.8)	0.772
Kiambu	138 (51.5)	153 (50.7)	291 (51.2)	

*Multiple response option.

IUD, intrauterine device.

### Feasibility for e-commerce pharmacy provision of LNG 1.5 mg for pericoital use

A total of 2291 doses of LNG 1.5 mg were dispensed to 300 women over a 12-month period (e-commerce pharmacy sales data; table 2). Most women (83%) used USSD to obtain the study pill. More than half (52%) preferred obtaining 5–6 doses per order and more than a third (37%) obtained the study pill more than once. Of the 266 women who obtained the study pill and completed the extended survey, most (83%) were satisfied/very satisfied with e-commerce platform services, citing convenience and ease of use. In addition, most (76%) women felt that Kasha’s staff were supportive/friendly.

**Table 2:** Compliance/adherence, satisfaction and the likelihood of continued use of levonorgestrel 1.5 mg for pericoital use

Parameter	n	%
How participants ordered the study pill (n=300)*		
USSD	249	83.0
Website	48	16.0
Call centre	3	1.0
Doses dispensed (total)	2291	
Doses obtained at a time (units) (n=565)†		
1–2	199	35.2
3–4	75	13.3
5–6	291	51.5
Proportion of women who obtained study pill more than once (n=300)*	110	37.0
Experience talking to the e-commerce pharmacist (n=266)		
Supportive/friendly	201	75.6
Not supportive (harsh, judgmental)	39	14.7
Do not know/no answer	26	9.8
Satisfaction with e-commerce pharmacy services (n=201)§		
Very satisfied	100	49.8
Satisfied	66	32.8
Not satisfied/would not order again	35	17.4
Reason for being very satisfied/satisfied (n=166)‡		
It is convenient/easy to use	151	91.0
It is dependable (ie, I can call them if there is a problem)	46	27.7
It is discreet	34	20.5
I dislike visiting a pharmacy	20	12.0
Other (specify)	61	36.7
Days having sex per month (n) (n=266)		
1–2	90	33.8
3–5	142	53.4
6+	34	12.8
Using study pill every time having sex (n=266)		
Every time	212	79.7
Most of the time	35	13.2
Half the time	12	4.5
Infrequently/never	7	2.6
Average number of times per month used the study pill	1.3	
Time of use (before sex)		
Always before (n=266)	105	39.5
Within 6 hours before sex (n=105)	81	77.1
Average hours before sex (mean (SD))	4.9 (6)	
Time of use (after sex)		
Always after (n=266)	123	46.2
Within 6 hours after sex (n=123)	89	72.4
Average hours after sex (mean (SD))	4.1 (4)	
Time of use flexible (ie, sometimes before/after sex) (n=266)	35	13.2
Reasons for using study pill before sex (n=105)‡		
I like to be prepared	32	30.5
So I do not have to rush	5	4.8
It feels safer	50	47.6
So I will not forget	54	51.4
Other reasons	24	22.9
Reasons for using the study pill after sex (n=123)‡		
I do not want to use the product unless I know I need it	62	50.4
I usually do not know in advance when I am going to have sex	35	28.5
It is easier for me to remember	24	19.5
Other reasons	44	35.8
Would recommend LNG 1.5 mg for pericoital use to others (n=266)	222	83.5
Very satisfied/satisfied with LNG 1.5 mg for pericoital use (n=266)	250	94.0
Would ‘definitely’ continue using LNG 1.5 mg for pericoital use if it were available beyond study period (n=266)	205	77.1

*Includes 34 women who obtained the study pill but did not complete the survey.

†Total orders.

‡Multiple response option.

§Numbers do not add up to 266 due to missing cases.

LNG, levonorgestrel; USSD, Unstructured Supplementary Service Data.

### Acceptability of LNG 1.5 mg for pericoital use

The average length of time women remained in the study was 6.2 months. Therefore, we evaluated acceptability at the 6-month point. Most women reported having sex on average 3–5 times a month or fewer, which is consistent with the screening criteria (ie, six times or fewer a month). On average, women reported using the study pill 1.3 times per month. Most of the women reported using the study pill pericoitally, either within 24 hours before (average 4.9 hours) or after (average of 4.1 hours) coitus (table 2). Most women used the study pill pericoitally because they did not want to forget or wanted to use the pill only when in need. Of the 266 women who reported using pericoital LNG 1.5 mg pill at least once, 94% were satisfied or very satisfied, were willing to continue using it beyond the study period and would recommend it to others.

The timing of use varied by background characteristics (table 3). For example, women who used the method exclusively before sex were more likely to be older (aged 25 years or more), married or wanted no more children compared with those using the study pill after sex or flexibly.

**Table 3:** Timing of use by background characteristics

Characteristic	Always before sex (n=105) (n (%))	Always after sex (n=123) (n (%))	Sometimes before/after sex (n=35) (n (%))	P value
Age				
Younger women (18–24 years)	38 (36.2)	72 (58.5)	14 (40.0)	0.002
Older women (>24 years)	67 (63.8)	51 (41.4)	21 (60.0)	
Education attainment				
Primary or lower	15 (14.3)	6 (4.9)	6 (17.1)	0.023
Secondary or higher	90 (85.7)	117 (95.1)	29 (82.9)	
Marital status				
Married/has a boyfriend	49 (46.7)	33 (26.8)	14 (40.0)	0.007
Not in union	56 (53.3)	90 (73.2)	21 (60.0)	
Want another child				
Yes	77 (73.3)	112 (91.1)	29 (82.9)	0.002
No	28 (26.7)	11 (8.9)	6 (17.1)	
Satisfaction with pericoital LNG 1.5 mg pill				
Satisfied	66 (62.9)	71 (57.7)	24 (68.6)	0.461
Not satisfied	39 (37.1)	52 (42.3)	11 (31.4)	
Likelihood of future use of pericoital LNG 1.5 mg pill				
Would use the study pill again	77 (73.3)	98 (79.7)	28 (80.0)	0.478
Would not use	28 (26.7)	25 (20.3)	7 (20.0)	
If you were to find out that you were pregnant today, would this be a problem?				
Yes, a big problem	82 (78.1)	94 (76.4)	25 (71.4)	0.723
Not a big problem	23 (21.9)	29 (23.6)	10 (28.6)	

LNG, levonorgestrel.

### Safety

There were 355 incidences of side effects reported by 147 women during the bimonthly surveys. The most reported side effect was nausea followed by headache, changes in bleeding pattern, cramps and dizziness. All side effects were reported as mild and tolerable (online supplemental figure 1).

10.1136/bmjsrh-2022-201653.supp1 Supplementary data

## Discussion

Over the past decade, there has been a call for developing and deploying an ‘on-demand’ pericoital pill for women having infrequent sex.^20–22^ The rationale is that pericoital methods will directly address women’s unmet FP needs and potentially increase aggregate mCPR. This study adds to the body of evidence about the feasibility of e-commerce provision and acceptability of the LNG 1.5 mg for pericoital use among women having infrequent sex.

Accessing LNG 1.5 mg for pericoital use through an e-commerce platform is feasible and offers women convenience, privacy and a home delivery option. The e-commerce pharmacy platform met pericoital LNG 1.5 mg pill adopters’ needs and further bolstered the rationale for providing the pill through pharmacies and digital platforms. Digital technologies and platforms can improve access to health information and services by removing or reducing barriers such as inconvenient service locations and hours of service or loss of privacy.^23^ Most adopters preferred to use USSD to access the study pill, perhaps due to ease of use or familiarity with the technology. Thus, different market segments might need different levels of support to access health products through non-traditional service delivery methods. Most women found the e-pharmacy platform convenient and easy to use, were satisfied with telephone-based counselling, and could follow instructions on how to use the pericoital LNG 1.5 mg pill to prevent pregnancy.

Most women used the study pill within 24 hours before/after sexual exposure thus taking advantage of the pericoital nature of the LNG 1.5 mg. At least 40% of women exclusively used the study method within 24 hours before sex (the majority within 6 hours). Thus, women can distinguish between pericoital LNG 1.5 mg pill and postcoital EC pills. Our finding addresses the doubts among providers and FP stakeholders about the lack of distinctions between these two methods among users.^21^ Furthermore, the availability of the pericoital LNG 1.5 mg pill will not increase sexual or contraceptive risk-taking behaviour—most women used fewer than six doses in a month, suggesting that fears of overuse floated in policy and programmatic circles may be unfounded.

Most adopters were satisfied or very satisfied with LNG 1.5 mg for pericoital use, were willing to continue using and would recommend it to others. Moreover, more than a third (37%) of adopters obtained the study pill more than once, suggesting potential demand and acceptability of a pericoital FP method. Our findings are consistent with a previous study that showed high acceptability of LNG 1.5 mg for pericoital use in multiple countries: Hungary, Thailand, Singapore and Brazil.^10^ Furthermore, the study pill appeals to women having infrequent sex. Of the 266 women who completed the survey, 80% used pericoital LNG 1.5 mg every time they had sex and used fewer than the allowable maximum doses (ie, six pills per month). More than half of the women reported experiencing side effects and changes in bleeding patterns; however, these were mild and tolerable similar to previous studies which noted insignificant influence of side effects on satisfaction and frequency of use of the LNG 1.5 mg pill.^9 10^

LNG 1.5 mg for pericoital use could potentially address the unmet need for FP; 44% of adopters had never used a more effective modern method (ie, IUD, injectable, implant, daily pill) while 11% had never used any method prior to enrolment in the study. Furthermore, women who had previously discontinued using modern methods were willing to use the LNG 1.5 mg pericoital pill as their primary contraceptive method. Our finding reflects the pericoital LNG 1.5 mg pill’s potential to fill the unmet FP need among women having infrequent sex or those dissatisfied with other modern methods. Therefore, the government should consider introducing the pericoital LNG 1.5 mg pill to expand FP method choice.^21^

The study has some limitations. Several other factors might have contributed to the high number of participants who despite enrolling initially, were no longer interested in the study, and hence discontinued or remained inactive (ie, non-adopters). All participants were enrolled remotely, and data collection was telephone-based without face-to-face contact (due partly to the COVID-19 pandemic). For this reason, we posit that some women became reluctant to participate, lacked confidence, or were concerned about taking a new contraceptive pill to protect against a real risk (ie, pregnancy). In addition, e-commerce provision, while beneficial in many ways, might have been imperfect (and new) for some participants. We interviewed women exiting the study; some participants became disinterested because they had infrequent sex and were not planning to engage in sexual activity during the remaining study period. Some participants, especially those without experience with the study pill, were demotivated by the frequent bimonthly telephone calls and therefore stopped picking up research assistants’ telephone calls or blocked the study lines. Similar challenges have been documented in other telephone-based surveys.^24^ Finally, the study did not have a control group, and data on use and safety (ie, side effects) were self-reported without a robust clinical assessment.

## Conclusions

E-commerce pharmacy provision of LNG 1.5 mg for pericoital use is feasible and a viable option for improving access to FP information and services. A pericoital LNG 1.5 mg pill is acceptable among women having infrequent sex, including those who have never used, are dissatisfied, or discontinued other modern methods. In addition, pericoital LNG 1.5 mg pill adopters experienced tolerable side effects, many of whom were satisfied with this method.

## Data Availability

Data are available upon reasonable request. Requests to access the data may be sent to Population Council, Dataverse, email: publications@popcouncil.org for information on data access.
