# Supplementary figures and images for: Feasibility of e-commerce pharmacy provision and acceptability of levonorgestrel 1.5 mg for pericoital use in urban and peri-urban settings in Kenya: a prospective cohort study
Source: BMJ Sex Reprod Health. 2022 Nov 2;49(1):35–42. doi: 10.1136/bmjsrh-2022-201653 (PMC9872235; doi:10.1136/bmjsrh-2022-201653)

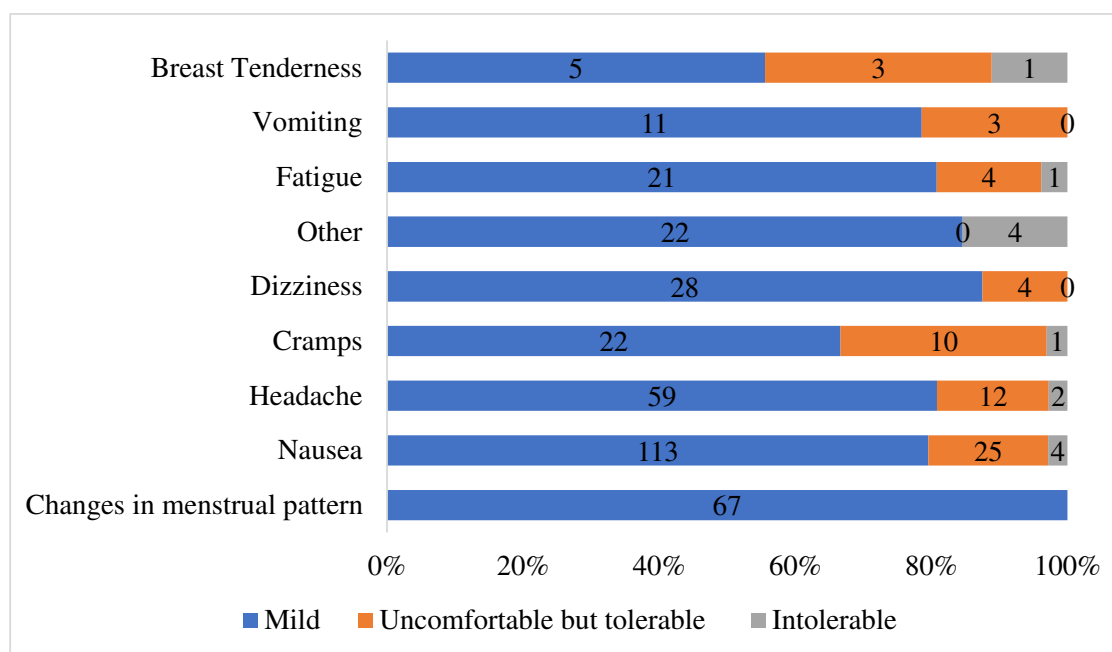

**Supplementary Figure 1: Self-reported side effects**

Supplement: Supplementary data [file bmjsrh-2022-201653supp001.pdf]
